# First person – Zakia Abdelhamed

**DOI:** 10.1242/dmm.047852

**Published:** 2020-10-30

## Abstract

First Person is a series of interviews with the first authors of a selection of papers published in Disease Models & Mechanisms, helping early-career researchers promote themselves alongside their papers. Zakia Abdelhamed is first author on ‘A novel hypomorphic allele of *Spag17* causes primary ciliary dyskinesia phenotypes in mice’, published in DMM. Zakia is a research associate in the lab of Dr Rolf Stottmann at Cincinnati Children's Hospital Medical Center, Cincinnati, OH, USA, investigating animal models that recapitulate a human condition for understanding disease pathogenesis.


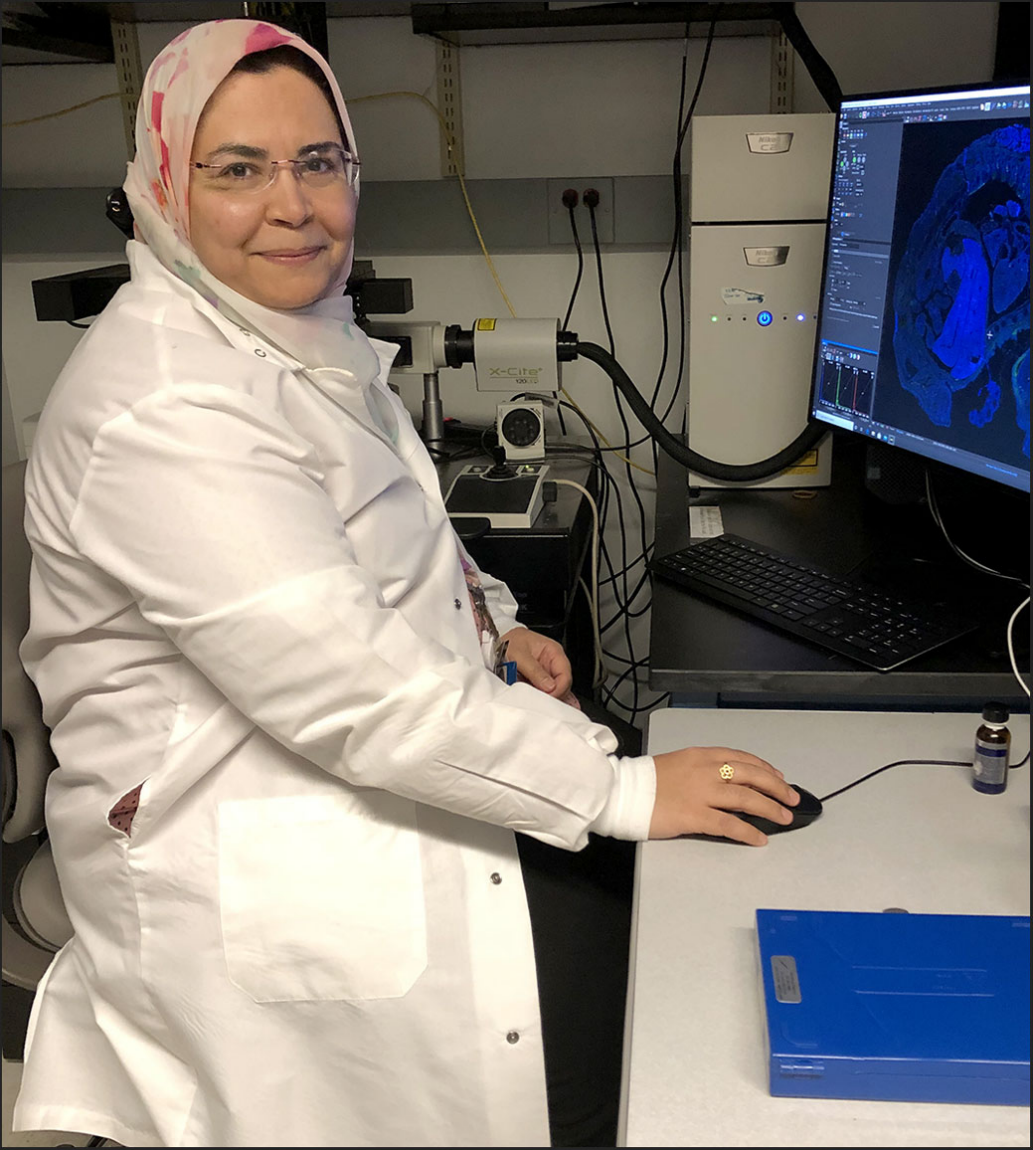


**Zakia Abdelhamed**

**How would you explain the main findings of your paper to non-scientific family and friends?**

Our genetic screen discovered an interesting new mouse model of human primary ciliary dyskinesia (PCD), the *Spag17^Pcdo^* mouse. *Spag17* is a motile cilia gene known to have a prominent role in the development of the central structure of the motile cilia. In addition, *Spag17* has also been reported to have a role in development and elongation of the long bone. Different mutations cause different presentations in human patients. The *Spag17^Pcdo^* mutants we report in our study developed hydrocephalus (water on the brain), mucus accumulation in the lung and male infertility due to total lack of development of sperm flagellum (cilium), but no bone-related malformation. Our data indicate that *Spag17^Pcdo^* is a specific mutation that affects motile cilia development, but no other structures in the developing embryo. This makes it a very unique model of particular importance to study the pathogenesis of human PCD.

“[…] a fellow or trainee who proves themselves as a good asset should be given the chance to transition to the next career level in their home institution if they wish to stay.”

**What are the potential implications of these results for your field of research?**

Defective development of any of the motility structures of the motile cilia results in a specific beating abnormality and a subcategory of primary ciliary dyskinesia in human patients. For example, inner dynein arm mutants usually have the most severe motile cilia beating abnormalities, and they are easily identified during clinical testing and patient diagnosis. However, central pair mutants produce very subtle beating defects, no organ laterality and milder overall patient manifestations. Thus, this group of patients is the hardest to diagnose and to assign to a subcategory of PCD. Our data present to the scientific community of cilia and ciliopathies a new model of central pair structure mutants. We believe that further characterization of this model can lead to discovery of a better diagnostic test to accelerate diagnosis of this subcategory of PCD.

**What are the main advantages and drawbacks of the model system you have used as it relates to the disease you are investigating?**

Mouse models with mutation in any of the motile ciliopathy genes, including *Spag17^Pcdo^*, recapitulate the human condition, so are useful for understanding the molecular mechanisms of disease pathogenesis. Drawbacks include the development of extra phenotypes or a more penetrant phenotype, such as hydrocephalus, which in animal models of PCD appear earlier in development and with higher penetrance. This differs from humans, in whom hydrocephalus has a much lower frequency of presentation in PCD patients. The hydrocephalus is almost always very severe in mouse models of PCD and led to premature death of the animals, which hinders characterization of the other phenotypes, such as the respiratory phenotypes during later developmental stages. This could be explained by differences between mouse and human brain anatomy. Nevertheless, the data obtained from characterizing the hydrocephalus mutant mice models are still valid in explaining the mechanisms involved in the human hydrocephalus disease pathogenesis.

**What has surprised you the most while conducting your research?**

The difference in SPAG17 protein expression between different tissues that carry the mutant allele. Also, the hyperkinetic mutant cilia in the *Spag17^Pcdo^* mutant forebrain, and the occurrence of severe intraventricular hemorrhage in less than 10% of the *Spag17^Pcdo^* mutant animals.

**Describe what you think is the most significant challenge impacting your research at this time and how will this be addressed over the next 10 years?**

Availability of funding for postdoctoral fellows and research associates. This is especially acute for the community of international scholars.

**Figure:**
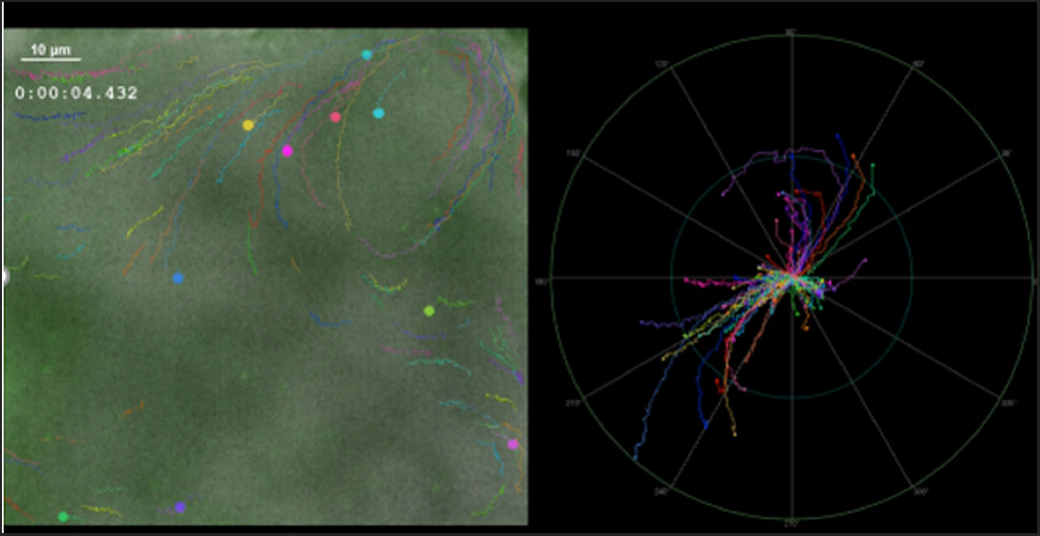
**A still image of high-speed video microscopy recordings of the aqueduct in the *Spag17^Pcdo^* mutants, with different colors representing fluorescent microbead tracks pointing in different directions (left). A polar graph showing a graphic representation of the abnormal directionality of the flowing beads in the *Spag17^Pcdo^* mutant aqueduct (right).**

**What changes do you think could improve the professional lives of early-career scientists?**

More involvement of postdoctoral fellows and research associates in grant writing. Institutional support in addition to PI support during the transition from a postdoctoral to a faculty position. Most institutions prefer the external applicant when hiring for a faculty, and there is a general rule that most postdoctors have to leave for their faculty position, which is not always an option for many of the postdoctors who are looking for early-career positions. I hope institutions can be more selective with this rule; a fellow or trainee who proves themselves as a good asset should be given the chance to transition to the next career level in their home institution if they wish to stay.

**What's next for you?**

Establishing my own research program in cilia and ciliopathies.
